# A Multicenter Cohort Study in Patients With Primary Empty Sella: Hormonal and Neuroradiological Features Over a Long Follow-Up

**DOI:** 10.3389/fendo.2022.925378

**Published:** 2022-06-23

**Authors:** Giulia Carosi, Alessandro Brunetti, Alessandra Mangone, Roberto Baldelli, Alberto Tresoldi, Giulia Del Sindaco, Elisabetta Lavezzi, Elisa Sala, Roberta Mungari, Letizia Maria Fatti, Elena Galazzi, Emanuele Ferrante, Rita Indirli, Emilia Biamonte, Maura Arosio, Renato Cozzi, Andrea Lania, Gherardo Mazziotti, Giovanna Mantovani

**Affiliations:** ^1^ Fondazione IRCCS Ca’ Granda Ospedale Maggiore Policlinico, Endocrinology Unit, Milan, Italy; ^2^ Sapienza University of Rome, Department of Experimental Medicine, Rome, Italy; ^3^ Humanitas University, Department of Biomedical Sciences, Pieve Emanuele, Italy; ^4^ IRCCS Humanitas Research Hospital, Endocrinology, Diabetology and Andrology Unit, Rozzano, Italy; ^5^ University of Milan, Department of Clinical Sciences and Community Health, Milan, Italy; ^6^ A.O. San Camillo Forlanini, Endocrinology Unit, Department of Oncology and Medical Specialities, Rome, Italy; ^7^ Humanitas Gavazzeni, Endocrinologia, Bergamo, Italy; ^8^ IRCCS Istituto Auxologico Italiano, Department of Endocrine and Metabolic Diseases, Milan, Italy; ^9^ Niguarda Hospital, Division of Endocrinology, Milan, Italy

**Keywords:** empty sella, sella turcica, pituitary, hypopituitarism, hypogonadism, neuroendocrinology, intracranial hypertension, traumatic brain injury

## Abstract

**Objective:**

primary empty sella (PES) represents a frequent finding, but data on hormonal alterations are heterogeneous, and its natural history is still unclear. Our aim was to evaluate the pituitary function of patients with PES over a long follow-up.

**Design:**

multicenter retrospective cohort study enrolling patients referred between 1984-2020 to five Pituitary Units, with neuroradiological confirmed PES and a complete hormonal assessment.

**Methods:**

we analyzed hormonal (including basal and dynamic evaluations), clinical and neuroradiological data collected at diagnosis and at the last visit (at least 6 months of follow-up).

**Results:**

we recruited 402 patients (females=63%, mean age=51.5 ± 16 years) with PES (partial, total, undefined in 66%, 13% and 21%, respectively). Hypopituitarism was present in 40.5% (hypogonadism=20.4%, hypoadrenalism=14.7%, growth hormone deficiency=14.7%, hypothyroidism=10.2%, diabetes insipidus=1.5%; multiple deficiencies=11.4%) and hypeprolactinemia in 6.5%. Interestingly, hormonal alterations were diagnosed in 29% of incidental PES. Hypopituitarism was associated with male sex (*p*=0.02), suspected endocrinopathy (*p*<0.001), traumatic brain injury (*p*=0.003) and not with age, BMI, number of pregnancies and neuroradiological grade. A longitudinal assessment was possible in 166/402 (median follow-up=58 months). In 5/166 (3%), new deficiencies occurred, whereas 14/166 (8.4%) showed a hormonal recovery. A progression from partial to total PES, which was found in 6/98 patients assessed with a second imaging, was the only parameter significantly related to the hormonal deterioration (*p*=0.006).

**Conclusions:**

this is the largest cohort of patients with PES reported. Hypopituitarism is frequent (40%) but hormonal deterioration seems uncommon (3%). Patients need to be carefully evaluated at diagnosis, even if PES is incidentally discovered.

## Introduction

The term “empty sella” (ES) refers to the herniation of the subarachnoid space within the sella turcica, with consequent flattening of the pituitary gland at its base (1). ES is classified as primary empty sella (PES) when unrelated to any known pituitary disorder, and secondary empty sella (SES) when it results as a sequela of a pituitary injury, such as the spontaneous necrosis of a pituitary adenoma, an infective or autoimmune process or a previous surgical, pharmacological or radiotherapy treatment. Therefore, we distinguish between a primary and secondary origin according to anamnestic data. Radiological features of PES and SES are generally similar. In some cases, the finding of a sellar bony expansion could be suggestive for a prior existing space occupying mass but bony enlargement has also been reported in presumed primary ES (2). Several hypotheses have been proposed regarding PES pathogenesis, including congenital sellar diaphragm incompetence, an increase in intracranial cerebrospinal fluid (CSF) pressure, or a variation in pituitary volume (3, 4), yet to now, it remains unclear. Nevertheless, studies have described obesity, hypertension, and pregnancies as possible risk factors of PES (2, 4–6). It is mostly described in women, with a peak incidence at 30-40 years (2, 4).

The prevalence of ES ranges from 6 to 20% in autopsy studies and also in clinical practice it is commonly detected, being reported in around 8–35% of the general population, with an increasing incidence in the last few decades possibly due to the improvements and availability of neuroimaging techniques (up to 38% in MRI studies) (7–10). ES is a radiological diagnosis that requires a CT/MRI scanning to be performed. It is referred to as partial ES when the sella turcica is filled with CSF to a level of <50%, and complete ES when >50% of the sella is filled with CSF and pituitary tissue is ≤ 2 mm (6, 11). Although PES is often an incidental finding in asymptomatic patients, a variable degree of neurological, visual, and/or endocrine disorders have been described. The pooled prevalence of hypopituitarism is estimated between 19 and 68%, so that hormone evaluation is always recommended at diagnosis. Multiple axis dysfunctions are more commonly described than isolated pituitary insufficiency. According to current evidence, the somatotropic axis is the most frequently affected, followed by the gonadotropic axis. Mild hyperprolactinemia is also often described in PES (2, 4, 8, 9, 11–13).

Many authors suggest performing a re-evaluation of patients with PES after 24 to 36 months because of the theoretical risk of progression (4, 6, 9). However, although several series of patients with PES have been reported in the literature (2, 4, 11–15), to our knowledge data on the natural history of PES and on the possible disease progression over time are still lacking. Among the largest series reported, De Marinis et al. described 213 cases of PES, detecting documented endocrine abnormalities in 19% of them (including hyperprolactinemia; 8% if including deficiencies only). Besides for 21 patients that underwent surgery, a follow-up description was available for 98 cases, and only one patient whose pituitary function was normal at baseline developed hypopituitarism (4). Guitelman and colleagues provided the baseline picture of 175 patients and detected some degree of hypopituitarism in 28%; however, no follow-up data could be obtained (2). Therefore, there are no clear evidence-based recommendations on the proper management of patients with PES.

The aim of this study was to analyze the characteristics and the natural history of a large cohort of patients with PES providing a long-term follow-up, and possibly to derive some recommendations for the clinical management of these patients. In order to do so, we performed a retrospective observational cohort study of 402 patients with PES referring to four tertiary endocrinology units (Fondazione IRCCS Ospedale Maggiore Policlinico, Milano; Humanitas Research Hospital, Milano; IRCCS Istituto Auxologico Italiano, Milano; Azienda Ospedaliera San Camillo, Roma; ASST Grande Ospedale Metropolitano Niguarda). Follow-up data were obtained for 166 of these patients, with a median follow-up of 58 months.

## Materials and Methods

This is a retrospective, longitudinal, multicenter study based on medical records of 402 patients (255 females, 147 males; mean age 51.5 ± 15.5 years, range: 13-84) with ES attending four Pituitary Units in the period between 1984 and 2020. The inclusion criteria were: 1) magnetic resonance imaging (MRI) diagnosis of ES; 2) availability of hormonal data at baseline; 3) written informed consent. Previous pituitary surgery and irradiation were considered exclusion criteria. In only six patients referred before 1995, PES was diagnosed by computed tomography but they have been all reassessed with an MRI scan.

The primary aim of this study was to evaluate the prevalence and determinants of hypopituitarism at diagnosis of ES. As secondary aims, we explored the incidence and determinants of newly developed pituitary hormone deficiencies after at least a 6-month follow-up. These end-points were addressed by a retrospective review of laboratory findings and clinical charts of the patients. The following clinical and biochemical data were collected: age at diagnosis of ES, sex, body mass index (BMI), reasons for performing MRI, the entity of ES, history of traumatic brain injury, number of pregnancies in women, results of evaluations of pituitary hormone secretion at baseline, duration of follow-up whenever performed, the outcome of pituitary function in patients followed-up at the Pituitary Units.

Patients with pituitary thickness ≤2 mm and more than 50 % filled with cerebrospinal fluid (CSF) were considered as total ES; patients with pituitary thickness ≥3 mm but ≤7 mm (the mean diameter of the normal pituitary gland in adults) and less than 50 % filled with CSF were considered as partial ES (4, 9).

Hormonal evaluations included: morning serum corticotropin (ACTH), cortisol, thyrotropin (TSH), free-thyroxine (FT4), prolactin (PRL) after 60’ resting period, insulin-like growth factor-1 (IGF1), random growth hormone (GH), LH, FSH, estradiol levels (in females), total testosterone levels (in males). In patients with cortisol levels between 3 and 15 µg/dl, stimulating tests with either ACTH or insulin tolerance test (ITTs) were performed (**Table 1**). Until 1998, the adrenal function was studied with a 250 μg Synacthen test. After 1998, the 1 μg Synacthen test was adopted as the routine test in patients with pituitary diseases. A cortisol peak of 18 µg/dl was used in these tests for excluding adrenal insufficiency, according to international guidelines (16, 17). From 2017, a cortisol cut-off of 13.6 µg/dL was applied to the 1 μg Synacthen test in one out of five centers adopting the highly specific cortisol immunoassay Roche II (18).

**Table 1:** Demographical and clinical features of 402 subjects with empty sella at the study entry.

		*Cases evaluated*
Age (years)	51.50 ± 15.5	402
Sex (F/M)	255/147	402
BMI (Kg/m2)	28.18 ± 6.6	379
BMI categories		379
Thinnes	13 (3.4)	
Normal	112 (29.6)	
Overweight	139 (36.7)	
Obesity	115 (30.3)	
Entity of ES		321
Complete	54 (16.8)	
Partial	267 (83.2)	
Diagnosis of ES		402
Incidental	289 (71.9)	
Clinical suspicion of pituitary disease	113 (28.1)	
Traumatic brain injury	51 (23.1)	221
Number of pregnancies		229
0	62 (27.1)	
1	45 (19.7)	
2	85 (37.1)	
≥3	37 (16.2)	
Stimulating tests for cortisol		326
Low-dose-ACTH test	176 (54.0)	
Standard-dose-ACTH test	125 (38.3)	
ITT	25 (7.7)	
Stimulating tests for GH		108
GHRH+arginine	41 (38.0)	
ITT	65 (60.2)	
Not specified	2 (1.8)	
Pituitary hormone deficiencies		402
None	239 (59.5)	
1 deficiency	117 (29.1)	
≥2 deficiences	46 (11.4)	
Hyperprolactinemia	25 (6.5)	386

Categorical data were presented as n/n or n (%), whereas continuous data were presented as mean ± SD.

F, females; M, males; BMI, body mass index; ES, empty sella; ACTH, corticotropin hormone; ITT, insulin tolerance test; GH, growth hormone; GHRH, growth hormone releasing hormone.

Central hypothyroidism was diagnosed in the presence of low free thyroxine levels associated with low or normal thyroid-stimulating hormone values. Central hypogonadism in men was defined by low total testosterone levels associated with normal or low gonadotropins (luteinizing hormone, follicle-stimulating hormone) in women of fertile age by clinical findings (amenorrhea or oligomenorrhea) associated with low 17-beta estradiol levels and low or normal gonadotropins and in postmenopausal women by the absence of high follicle-stimulating hormone levels (19). Diagnosis of growth hormone deficiency (GHD) was made by performing stimulating tests with either GHRH plus Arginine or ITT (**Table 1**). In subjects evaluated by GHRH plus arginine, severe GHD was diagnosed by GH peak lower than 9 µg/L when BMI was less than 30 kg/m2 and 4.0 µg/L for BMI equal or greater than 30 kg/m^2^. In patients evaluated by ITT, severe GHD was diagnosed by GH peak < 3 µg/L. In patients with low IGF-1 and three other documented pituitary hormone deficits, GHD was diagnosed without performing stimulating tests (16, 20, 21). To assess diabetes insipidus (DI), patients were evaluated for serum sodium, plasma and urine osmolarity, and liquid balance. DI was defined as new-onset emission of high volume (>40 ml/kg/day) of hypotonic urine (<300 mOsm/Kg) in absence of hyperglycemia and hyponatremia (22).

The study was approved by the Ethical Committees of the IRCCS Humanitas Research Hospital, Rozzano (MI). The patients gave consent to use their clinical data for research purposes.

### Statistical Analysis

Normally and non-normally distributed variables were reported as mean ± standard deviation and median and absolute ranges, respectively. T-test and non-parametric tests (i.e., Mann-Whitney’s and Kruskall Wallis’ tests) were used for the comparisons in normally and non-normally distributed variables, respectively. Determinants of hypopituitarism at study entry and at the follow-up were explored with logistic regression analysis. All risk factors with a P value under 0.10 were then submitted to a backward multivariate logistic regression analysis to define the independent determinants of hypopituitarism at study entry. A P value less than 0.05 was considered significant.

## Results

**Table 1** shows the clinical features of 402 patients with ES enrolled in the study. In 113 patients (28.1%), the diagnosis of ES was guided by clinical and biochemical suspicion of pituitary disease, whereas in the remaining 289 patients (71.9%) the diagnosis of ES was incidental and MRI was performed because of neurological (*184 cases*), ophthalmological (*23 cases*), otolaryngological (*14 cases*) symptoms or for other non-specified reasons (*68 cases*).

Patients with an incidental diagnosis of ES were more frequently males as compared to those in whom the diagnosis of ES was guided by clinical and biochemical suspicion of pituitary disease (**Table 2**).

**Table 2:** Features at study entry of subjects with incidental diagnosis of empty sella (ES) as compared to those in whom the diagnosis was guided by clinical suspicion of pituitary disease.

		INCIDENTAL DIAGNOSIS OF ES		P-values
	Cases evaluated	YES	NO	
Age (years)	402	51.3 ± 15.5	50.5 ± 15.3	0.761
Sex (F/M)	402	211/78	44/69	< 0.001
BMI (Kg/m2)	379	28.1 ± 6.8	28.2 ± 6.0	0.457
Entity of ES (Partial/Complete)	321	187/36	80/18	0.624
Traumatic brain injury	221	39 (24.5)	12 (19.4)	0.412
Central hypogonadism	402	25 (8.7)	57 (50.4)	< 0.001
Central hypothyroidism	402	8 (2.8)	33 (29.2)	< 0.001
Central Adrenal insufficiency	326	39 (13.5)	20 (17.7)	0.210
GHD	402	36 (12.5)	23 (20.4)	0.044
Diabetes insipidus	402	5 (1.7)	1 (0.9)	1
Hyperprolactinemia	386	8 (2.9)	17 (15.7)	< 0.001

Categorical data were presented as n/n or n (%), whereas continuous data were presented as mean ± SD.

ES, empty sella; F, females; M, males; BMI, body mass index; ACTH, corticotropin hormone; GHD, growth hormone deficiency.

Traumatic brain injury was reported by 51 out of 221 patients (23.1%) evaluated for this issue. The prevalence of partial and total PES was not different in patients with and without a TBI (partial PES in 78 vs 74%, respectively, P=0.68). Among the 379 patients in whom BMI values were available, 112 patients (29.6%) had normal BMI, whereas thinness, overweight, and obesity were found in 13 (3.4%), 139 (36.7%), and 115 (30.3%) cases, respectively. In women with available information on the number of pregnancies (*229 cases*), 62 (27.07%) were nulliparous, 45 (19.65%) referred one pregnancy, 85 (37.11%) two pregnancies, and 37 (16.16%) three or more pregnancies. ES was total in 54 patients, partial in 267, and not defined in the remaining 81/402 patients.

No significant differences in age, BMI, traumatic brain injury BMI and entity of ES were found between patients with incidentally diagnosed ES and those in whom the diagnosis was guided by clinical suspicion of pituitary disease (**Table 2**).

### Prevalence and Determinants of Pituitary Hormone Alterations at Diagnosis of ES

At the study entry, 163 out of 402 patients (40.5%) had one or more pituitary hormone deficiencies (single in 118 cases, multiple in 46 cases) (**Table 1**). Central adrenal insufficiency, hypothyroidism, hypogonadism, GHD and DI were found in 59 (14.7%), 41 (10.2%), 82 (20.4%), 59 (14.7%) and 6 (1.5%), respectively. Mild hyperprolactinemia was reported in 25 cases (6.5%).

**Figure 1:**
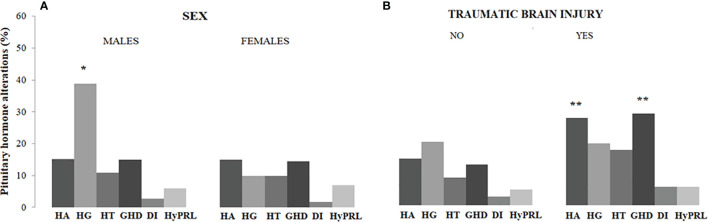
Prevalence of pituitary hormone alterations in male and female patients with PES **(A)** and in patients with and without a history of traumatic brain injury **(B)**. HA, hypoadrenalism; HG, hypogonadism; HT, hypothyroidism; GHD growth hormone deficiency; DI, diabetes insipidus; HyPRL, hyperprolactinemia; * and **, statistically significant.

Hypothyroidism, hypogonadism, GHD, and hyperprolactinemia were significantly more frequent in patients with a diagnosis of ES guided by clinical suspicion of pituitary disease as compared to those in whom the diagnosis of ES was incidental, without significant differences in hypoadrenalism and DI (**Table 2**). Males had a higher prevalence of central hypogonadism (P<0.001) as compared to females, whereas subjects with a history of traumatic brain injury had a higher prevalence of central hypoadrenalism (P=0.049) and GHD (P=0.006) as compared to those who did not refer brain injury (**Figure 1**).

In the multivariate logistic regression analysis, hypopituitarism was significantly and independently associated with male sex (odds ratio [OR] 2.05, 95% confidence interval [C.I.] 1.12-3.73; P=0.019), clinical suspicion of pituitary disease (OR 4.27, 95% C.I. 2.19-8.34; P<0.001) and previous traumatic brain injury (OR 2.80, 95% C.I. 1.41-5.57; P=0.003) (**Table 3**). In a secondary analysis performed in the male group, we did not find a significant relationship between BMI values and the presence of hypogonadism (P=0.12).

**Table 3:** Results of univariate and multivariate logistic regression analysis assessing the determinants of pituitary hormone deficiency at study entry in subjects with empty sella.

		UNIVARIATE ANALYSIS			MULTIVARIATE ANALYSIS		
Covariates	N. cases	OR	95% C.I.	P-values	OR	95% C.I.	P-values
Age	402	1.00	0.99-1.01	0.882			
Male sex	402	2.71	1.78-4.12	< 0.001	2.05	1.12-3.73	0.019
BMI	379	0.99	0.97-1.03	0.830			
Clinical suspicion	402	6.02	3.73-9.71	< 0.001	4.27	2.19-8.34	< 0.001
Traumatic brain injury	221	2.25	1.19-4.26	0.013	2.80	1.41-5.57	0.003
N. of pregnancies	229	1.04	0.86-1.26	0.685			
Entity of ES	321	1.46	0.81-2.62	0.211			

BMI, body mass index; C.I., confidence interval; ES, empty sella; OR, odds ratio.

### Longitudinal Study

Among 166 patients followed-up for at least 6 months (median duration 58.3 months, range: 6.0-300.4), 5 (3.0%) developed one or more new pituitary hormone deficiencies (**Table 4**) and 14 (8.4%) showed a recovery of one or more pituitary insufficiency (**Table 5**). Interestingly, one patient of this group was treated with a CSF shunting for symptomatic idiopathic intracranial hypertension (see **Table 5**, patient number=10). She displayed an isolated secondary hypoadrenalism at diagnosis which was not confirmed on retesting after two years from treatment.

**Table 4:** Individual data of patients with empty sella who developed pituitary deficiency during the follow-up.

	Age (years)	Sex	Incidental diagnosis of ES	Follow-up (months)	NEW PITUITARY DEFICIENCIES AT FOLLOW-UP					MRI outcome of ES
					HA	HG	HT	GHD	DI	
1	50	F	Y	145,9	N	N	**Y**	N	N	**Impaired**
2	59	M	N	129,3	N	N	*NE*	**Y**	N	Stable
3	63	M	N	79,6	**Y**	N	**Y**	N	N	*NE*
4	55	F	N	64,7	N	N	N	**Y**	N	Stable
5	74	F	Y	82,3	**Y**	N	N	N	N	**Impaired**

F, females; DI, diabetes inspidus; ES, empty sella; GHD, growth hormone deficiency; HA, hypoadrenalism; HG, hypogonadism; HT, hypothyroidism; M, males; MRI, magnetic resonance imaging; N, NO; NE, not evaluated; Y, yes. Bold letters indicate patients who developed new deficiencies and showed an impairment of ES grade on MRI during follow-up.

**Table 5:** Individual data of patients with empty sella who recovered pituitary function during the follow-up.

N	Age (years)	Sex	Incidental diagnosis of ES	Follow-up (months)	RECOVERY OF PITUITARY FUNCTION DURING THE FOLLOW-UP				
					HA	HG	HT	GHD	DI
1	50	F	Y	26	Y	N	N	N	N
2	42	F	Y	22	Y	N	N	N	N
3	52	M	Y	35	Y	N	N	N	N
4	50	M	N	128	N	N	N	Y	N
5	50	F	N	100	N	N	N	N	N
6	71	F	Y	68	N	N	N	N	N
7	38	M	Y	61	Y	N	N	Y	N
8	52	M	Y	51	Y	Y	N	Y	N
9	60	M	N	30	N	Y	N	N	N
10	41	F	Y	80	Y	N	N	N	N
11	28	M	Y	107	N	Y	N	N	N
12	65	F	Y	9	Y	N	N	N	N
13	50	F	Y	14	Y	N	N	N	N
14	31	F	Y	39	Y	N	N	N	N

F, females; DI, diabetes inspidus; ES, empty sella; GHD, growth hormone deficiency; HA, hypoadrenalism; HG, hypogonadism; HT, hypothyroidism; M, males; N, NO; Y, yes

Among 98 patients in whom MRI was longitudinally repeated, 6 patients (6.1%) showed a progression of ES from partial to total. In 2/6 there was a progression of hormonal deficiencies as well (see **Table 4**).

In the univariate regression analysis, the development of new pituitary deficiencies was significantly associated with impairment of ES entity (OR 22.5, 95% C.I 2.49-203.27; P=0.006), without significant association with age (P=0.151), sex (P=0.833), BMI (P=0.410), previous traumatic brain injury (P=0.999) and clinical suspicion of pituitary disease (P=0.165), duration of follow-up (P=0.532). No determinants of recovery of pituitary insufficiency were identified by univariate analysis (data not shown).

## Discussion

The prevalence of ES in clinical practice has been reported ranging from 8 to 35% in the general population, with an increasing incidence in the last few decades possibly due to the improvements and availability of neuroimaging techniques (7–10). This frequent finding thus poses an important clinical question on how to perform a correct endocrinological assessment at diagnosis and on how often to re-evaluate these patients. Here we present the largest cohort study and the first with a long follow-up in patients with PES. The previous series included smaller populations and follow-up data were mostly lacking.

Available data on hypopituitarism in PES are greatly variable, with rates ranging from 19 to 68% (2, 4, 8, 9, 11–13). In our series, hormonal deficiencies are present in 40% of patients. Only one series revealed a higher prevalence, probably due to its prospective design, which allowed systematic testing of all pituitary axes (11). The lowest rates have been described in studies where patients have been dynamically evaluated only in the presence of clinical suspicion of hypopituitarism (4). According to our data, we advise a complete endocrinological evaluation in PES at diagnosis. The majority of patients in our series displayed only one deficiency (72%) and central hypogonadism was the most frequent (20%), similarly to other studies, while other authors found GHD instead as the most common (4, 11, 12). These results are, again, probably related to the different use of dynamic tests. We found hypoadrenalism, hypothyroidism, and GHD equally present in about 15-20%. On the contrary, diabetes insipidus is very rare, confirming all existing data. Hyperprolactinemia is slightly lower than other series (6.5% vs 10-17%) (2, 4, 12, 13). The inclusion of a large group of incidental PES, where hyperprolactinemia is not frequent as expected, could explain this finding.

Patients with incidentally discovered ES are common in clinical practice (7) but few systematic data on pituitary function are available in this setting. Our study showed that hypopituitarism is present in a considerable proportion of incidental ES, especially central hypoadrenalism and GHD, even if not clinically overt. Therefore, a complete hormonal evaluation, including dynamic tests, is necessary for these patients as well, especially to unmask adrenal insufficiency, which is potentially life-threatening. Despite different opinions about when to administer GH replacement therapy to adults with GHD, we are in line with the international guidelines (16), which favor testing all patients and deciding individually who should be treated.

Numerous pituitary and extra-pituitary factors have been considered in the pathogenesis of PES. Some studies reported an association between PES and obesity (2, 4). Even if a causative link is not possible to be established, our data seem to confirm this finding [the rate of obese patients resulted higher than expected in the Italian population, 30.3% vs 10.9% (23)]. On the contrary, the number of multiple pregnancies was similar to the Italian data [53.1% of multiple pregnancies vs 55% in Italy (24)]. The history of TBI was collected in only 221/402 patients and, consequently, this data could be underestimated. However, we found TBI predictive of hypopituitarism, corroborating its potential role in the etiopathogenesis of PES. Besides TBI, the presence of clinical suspicion of endocrinopathy and male sex were further significant predictors. The first one is expected, while the second one is probably due to the high prevalence of hypogonadism in our group. This hormonal defect is more frequently diagnosed in males than in females in the general population (25). Moreover, whereas the prevalence of migraine is much greater in females than in males (ratio of 3:1) (26), female patients may have been more often investigated with a brain MRI during their lifetime, explaining the high proportion of incidental (and clinically silent) PES in this group.

Longitudinal results showed that only a few patients with PES change their hormonal profiles over time. On the opposite, some patients (14/166, 8.4%) displayed an improvement in pituitary function, especially in adrenal axes on retesting. We are aware that pitfalls in stimulation tests can explain why hormonal responses sometimes differ in the same patient (17, 27, 28). On the other hand, we can’t exclude a real recovery in the pituitary function. Interestingly, we described one patient who displayed a normalization in the hormonal assessment after correction of IH with a CSF shunting, highlighting the possible role of IH in the etiopathogenesis of empty sella and hypopituitarism. Over a median follow-up time of 58 months, which is the longest follow-up available in a cohort study on PES, only 5/166 patients (3%) showed hormonal deterioration. Among the assessed parameters, only the neuroradiological grade progression was related to the hormonal deterioration. Even if the group was small and no statistical analysis were feasible, 4/5 patients presented at least one hormonal deficiency at the time of diagnosis. In the same way, we could not establish an exact follow-up time needed to exclude an upcoming deterioration. According to our data, repeating MRI could be useful for identifying worsening patients (e.g. at least once, 12 months after diagnosis). According to these data, we recommend hormonal follow-up in all patients with hormonal deficiencies at diagnosis and/or showing a neuroradiological progression (e.g. basal exams every 12 months and individualized dynamic zre-evaluations). On the other hand, we advise clinical observation for patients without any pituitary impairment and with stable MRI.

The main limitation of the study is its retrospective design. Some data are lacking, especially on pituitary function (in particular, dynamic test for GH secretion available in only 27% of patients). The collection of anamnestic data was incomplete for some aspects (e.g. history of TBIs including sport related-brain injuries that have been related to the development of ES) (29). Some radiological features, such as the exact grade and the size of the sella turcica, have not been analyzed. Moreover, the study design did not allow us to perform some exploratory analyses which could be useful to clarify the etiopathogenesis of PES (e.g. autoimmune markers).

In conclusion, patients with primary empty sella need to be carefully evaluated at the time of diagnosis, even if PES is incidentally discovered. We suggest completing the assessment with dynamic tests (screening for GHD and adrenal insufficiency). Hypopituitarism is frequent (40%) but a deterioration in pituitary function seems uncommon (3%).

## Data Availability Statement

The raw data supporting the conclusions of this article will be made available by the authors, without undue reservation.

## Ethics Statement

The studies involving human participants were reviewed and approved by Ethical Committees of the IRCCS Humanitas Research Hospital, Rozzano (MI). The patients/participants provided their written informed consent to participate in this study.

## Author Contributions

GC, AL and GMan developed the original concept of this study and participated in the study design. All authors participated in data collection. GMaz, GC and AM conducted data analysis, interpretation and the writing of the original version of the manuscript. All authors participated in the manuscript revision. All authors have contributed to critical discussion and reviewed the final version.

## Funding

This work was supported by Ricerca Corrente funds from Fondazione IRCCS Ca’ Granda Ospedale Maggiore Policlinico and by grant NET-2018-12365454 from the Italian Ministry of Health.

## Conflict of Interest

The authors declare that the research was conducted in the absence of any commercial or financial relationships that could be construed as a potential conflict of interest.

## Publisher’s Note

All claims expressed in this article are solely those of the authors and do not necessarily represent those of their affiliated organizations, or those of the publisher, the editors and the reviewers. Any product that may be evaluated in this article, or claim that may be made by its manufacturer, is not guaranteed or endorsed by the publisher.
